# Evaluating the understandability and actionability of online CKD educational materials

**DOI:** 10.1007/s10157-023-02401-6

**Published:** 2023-09-16

**Authors:** Emi Furukawa, Tsuyoshi Okuhara, Hiroko Okada, Yuriko Nishiie, Takahiro Kiuchi

**Affiliations:** 1 Department of Health Communication, The University of Tokyo Graduate School of Medicine, 7-3-1 Hongo, Bunkyo-ku, Tokyo, 113-8655 Japan; 2 Department of Health Communication, School of Public Health, The University of Tokyo, Tokyo, Japan

**Keywords:** Chronic kidney disease, Health communication, Patient education

## Abstract

**Background:**

Previous studies have not fully determined whether online education materials on chronic kidney disease (CKD) for Japanese patients are easy to understand and help change their behavior. Therefore, this study quantitatively assessed the understandability and actionability of online CKD education materials.

**Methods:**

In September 2021, we searched Google and Yahoo Japan using the keywords “kidney,” “kidney disease,” “CKD,” “chronic kidney disease,” and “renal failure” to identify 538 webpages. We used the Japanese version of the Patient Education Materials Assessment Tool (PEMAT), ranging from 0 to 100%, to evaluate the understandability and actionability of webpages. We set the cutoff point to 70%.

**Results:**

Of the 186 materials included, the overall understandability and actionability were 61.5% (± 16.3%) and 38.7% (± 30.6%), respectively. The materials were highly technical in their terminology and lacked clear and concise charts and illustrations to encourage action. Compared to lifestyle modification materials on CKD overview, symptoms/signs, examination, and treatment scored significantly lower on the PEMAT. In addition, the materials produced by medical institutions and academic organizations scored significantly lower than those produced by for-profit companies.

**Conclusion:**

Medical institutions and academic organizations are encouraged to use plain language and to attach explanations of medical terms when preparing materials for patients. They are also expected to improve visual aids to promote healthy behaviors.

**Supplementary Information:**

The online version contains supplementary material available at 10.1007/s10157-023-02401-6.

## Introduction

Chronic kidney disease (CKD) is a growing epidemic, with 697.5 million persons worldwide experiencing kidney damage in 2017 [1]. In Japan, the number of adult patients with CKD is estimated to be 13.3 million, which means that one in eight people have CKD [2]. Studies have reported cardiovascular events, progression to end-stage renal disease, and early death as health risks for patients [3, 4]. Therefore, patients need to be able to detect CKD early and receive appropriate treatment through health checkups and follow-ups at medical institutions. To make this possible, the general public and patients must learn about CKD and its management [5–7].

However, several challenges are associated with health communication in patients with CKD. First, CKD is not well known to the public. A survey conducted in 2019 among the general public in Japan found that only 50.7% answered that they knew about CKD [8]. Second, many patients are unaware of their condition because CKD is asymptomatic until the advanced stage. In the National Health Survey conducted between 1999 and 2008, up to 90% of participants with 2–4 markers of CKD were unaware of the disease [9]. Third, inadequate health literacy is a concern for patient education in CKD, with 22.7% of patients with CKD reportedly having limited health literacy [10].

Previous studies have recognized that patient education is a necessary component of CKD care, with evidence that education increases patient knowledge, self-management, and activation across CKD stages [11, 12]. Approximately 70% of the Chronic Renal Insufficiency Cohort (CRIC) study participants reported using the Internet, email, and smartphones [13]. In addition, previous studies have shown that patients who collect health information online tend to make decisions based on it [14, 15].

Regarding CKD educational materials, an RCT in patients with advanced CKD showed that materials might support treatment selection [16]. However, it is questionable whether CKD educational materials can promote better health behaviors in the general public. Previous research using the Suitability Assessment of Materials (SAM) to analyze the CKD web materials found a need to improve message content, visuals/graphics, and layout/design [17]. In addition to assessing whether the material is suitable, The U.S. National Action Plan on Health Literacy lists the need to develop “actionable” health information as a key goal [18]. The Patient Education Materials Assessment Tool (PEMAT) systematically examines how the required action points are presented. The PEMAT was developed by experts under the direction of the Agency for Healthcare Research and Quality (AHRQ) and demonstrated content validity, internal consistency, and reliability [19]. A content analysis of online materials on CKD lifestyle modification using PEMAT reported that the “actionability” of the materials and the method of presenting visual aids need to be improved [20]. However, whether online CKD education materials in Japan help them understand and change patients’ behavior has not been fully discovered. In addition, the attributes and characteristics of “actionable” materials and the issues related to each topic of the materials have not been sufficiently examined. Therefore, using the PEMAT, this study aims to evaluate whether existing webpages with CKD educational materials are easy for patients and the general public to understand and encourage them to take action.

## Materials and methods

### Study design

This study used quantitative content analysis to review online CKD information systematically.

### Inclusion and exclusion criteria

We extensively searched for CKD education materials on September 30, 2021. The search involved obtaining patient educational materials distributed in clinical practice. Japanese websites providing educational information on CKD were included. The exclusion criteria were: (1) webpages aimed at medical professionals, (2) webpages containing misinformation, (3) patient experience, (4) webpages that are irrelevant (e.g., CKD in pets), (5) news or press releases, and (6) webpages not containing educational information. We selected materials from reliable sources that healthcare professionals would generally recommend. During the screening of the webpages, a board-certified nephrologist (EF) reviewed the information for reliability.

### Search strategy

We used a Japanese-language search string input into Google Japan and Yahoo Japan, which accounted for approximately 76% and 19% of all Internet searches in August 2021 [21]. We used Google Trends [22] to find the top five most frequently searched words related to CKD. The keywords “kidney,” “kidney disease,” “renal failure,” “CKD,” and “chronic kidney disease,” all of which were in Japanese, were entered into the search window, one keyword at a time. Because previous search engine analysis showed that 10–17% of users browse beyond the first three pages of the results [23], we reviewed 50 webpages per search engine for each term. To ensure comprehensiveness, we added online resources widely used in clinical practice. We performed an online search without an institutional login to avoid retrieving webpages that could only be seen with institutional credentials. To minimize bias in the search results, we logged out of our personal Google and Yahoo accounts and cleared our search history. We did not access content beyond the hyperlinks on the webpage under analysis.

### Variables extracted

EF classified the content as follows: (1) overview of CKD, (2) signs and symptoms, (3) screening and testing, (4) treatment options (e.g., medication and renal replacement therapies), and (5) lifestyle modification (e.g., healthy diet and exercise). We also classified the material upload sources into one of the following categories: (1) medical institutions, (2) academic institutions, (3) for-profit companies, (4) governmental organizations, and (5) non-profit organizations. We divided the target audience into two groups: (1) patients and their families and (2) the general public. The classifications were based on previous content analyses of online health resources [24, 25].

### Evaluation criteria

#### Understandability and actionability

The understandability and actionability of the material were rated using the Japanese version of the Patient Education Material Evaluation Tool for Printed Materials (PEMAT-P). PEMAT is intended for use by healthcare professionals, health librarians, and other professionals who provide health and medical information to patients and the general public [19, 26, 27]. As with the original PEMAT [19], the Japanese version of the PEMAT-P has been tested for reliability and validity [28]. In particular, it has proven predictive validity, which means that the assessment results are predictive of understandability and actionability for the audience. This instrument includes 23 items (16 on understandability and 7 on actionability) which were rated using a binary scale (agree = 1 or disagree = 0). We calculated the PEMAT-P scores by taking the sum of the points, dividing it by the total possible points, and multiplying the result by 100 to obtain a percentage. The author of the original version of PEMAT established the threshold for scores empirically at 70%[19]. Previous studies analyzing materials using PEMAT have followed this threshold [20, 29]. This study set the threshold of 70% to be considered understandable or actionable as well as to enable comparison of results with studies conducted in other countries.

#### Quality (natural flow and comprehensiveness)

The quality of each webpage was assessed using the Global Quality Score (GQS). GQS is used to assess the instructive aspects of online health resources for patients[30], which allows users to evaluate online health resources’ natural flow and comprehensiveness on a five-point Likert scale [31] (Supplementary file 1). It has been commonly used to evaluate health and medical information websites[32–34].

#### Readability

Since both PEMAT and GQS are subjective indicators, we additionally used readability, an objective measure to evaluate textual information. The text from each webpage was pasted to Microsoft Word (Microsoft Corp), and any formatting elements that might interfere with readability assessment (headings, symbols, author information, and references) were removed. The text was then assessed using jReadability [35]. This tool calculates readability based on the average length of sentences, difficulty level of words, proportion of grammatical parts of speech, and types of characters per sentence.

### Statistical analysis

We used descriptive statistics to summarize the characteristics and scores of the webpages. For PEMAT-P and jReadability, we performed a one-way analysis of variance (ANOVA) when comparing topics and sources. When significant differences were found, we performed Tukey’s multiple comparison test. For comparisons by the audience, we used an unpaired t-test. If there was a significant difference, we performed Kruskal–Wallis multiple comparisons, and we corrected p-values using the Benjamini–Hochberg method. For the GQS, we used the Kruskal–Wallis rank sum test when comparing topics and sources. If significant differences were found, we performed Kruskal–Wallis multiple comparisons. We corrected p-values using the Benjamini–Hochberg method. Two physicians (EF and YN) calculated inter-rater reliability (Gwet’s AC1) for a quarter of the webpages, since previous studies that analyzed the content of healthcare information usually analyzed inter-rater reliability by having two raters score the information. The two raters had a 60-min meeting in advance to review the PEMAT and GQS evaluation methods; for the PEMAT, User’s Guide [27] was used to provide details and examples of each item. A scale for inter-rater reliability ranges from poor to almost perfect. The scale is as follows: 0.00 to 0.20; Slight, 0.21 to 0.40; Fair, 0.41 to 0.60; Moderate, 0.61 to 0.80; Substantial, and 0.81 to 1.00; Almost Perfect [36].

P-values were two-sided, and *p* < 0.05 was considered statistically significant. All analyses were conducted using R version 4.0.3 (2020-10-10).

## Results

### Webpage selection

**Fig. 1:**
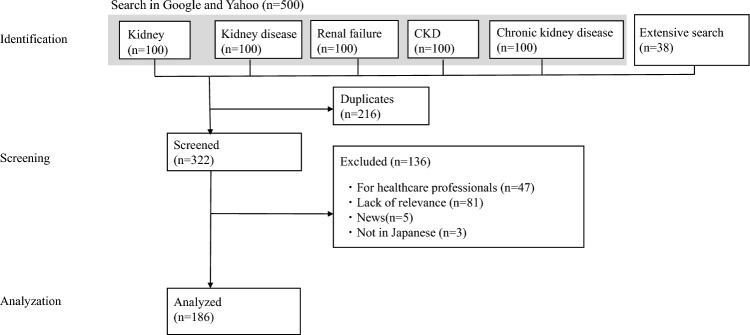
Selection of the webpages

Of the 537 webpages retrieved, we included 186 in the final analysis (Fig. 1). The webpage characteristics are summarized in Table 1. There was a substantial inter-rater agreement for PEMAT-P and GQS (average Gwet's AC1: 0.76 for PEMAT, 0.70 for GQS) (Supplementary file 2).

**Table 1:** Characteristics of the webpages included

	*n*	%
Source		
Governmental organization	19	10.2
Academic institution	17	9.1
Medical institution	64	34.4
Non-profit organization	18	9.7
For-profit company	68	36.6
Content		
Overview of CKD	104	55.9
Signs and symptoms	17	9.1
Screening and testing	13	7.0
Treatment options	22	11.8
Lifestyle modification	30	16.1
Target audience		
Patients and their families	135	72.6
General public	51	27.4

### Understandability and actionability

The mean understandability of these webpages was 61.5 (SD 16.3), and 57 (40%) webpages were acceptable for understanding. The highest understandability achieved was 100, and the lowest was 18. The mean actionability was 38.7 (SD 30.6), and 40 (21.5%) webpages were acceptable for actionability. The highest actionability achieved was 100, and the lowest was 0. Table 2 shows the results of the PEMAT-P ratings. Most webpages (*n* = 159, 88%) did not have a summary of the key points. In addition, many (*n* = 138, 74%) had difficulty using only common, everyday language and did not explain medical terms. Most webpages (*n* = 141, 76%) stated that the audience took at least one action. However, several webpages (*n* = 144, 77%) lacked visual aids to encourage the audience to take action.

**Table 2:** Score for each of the 23 PEMAT-P instrument criteria

Item #	Item	Materials rated “Agree = 1pt.”	
		*n*	%
Understandability			
Topic: content			
1	The material makes its purpose completely evident from the beginning	124	67
2	The material does not include information or content that distracts from its purpose	167	90
Topic: word choice & style			
3	The material uses common, everyday language	48	26
4	Medical terms are defined when they are used	98	53
Topic: use of numbers			
5	Numbers appearing in the material are clear and easy to understand	114	68
6	The material does not expect the user to perform calculations	154	83
Topic: organization			
7	The material breaks or “chunks” information into short sections	115	64
8	The material’s sections have informative headers	162	90
9	The material presents information in a logical sequence	174	94
10	The material provides a summary	22	12
Topic: layout & design			
11	The material uses visual cues (e.g., arrows, boxes, bullets, bold, larger font, highlighting) to draw attention to key points	100	54
Topic: use of visual aids			
14	The material uses visual aids whenever they could make content more easily understood (e.g., illustration of healthy portion size)	107	58
15	The material’s visual aids reinforce rather than distract from the content	117	74
16	The material’s visual aids have clear titles or captions	67	43
17	The material uses illustrations and photographs that are clear and uncluttered	88	56
18	The material uses simple tables with short and clear row and column headings	34	46
Actionability			
19	The material clearly identifies at least one action the user can take	141	76
20	The material addresses the user directly when describing actions	108	58
21	The material breaks down any action into explicit steps	42	23
22	The material provides a tangible tool (e.g., menu planners, checklists) whenever it could help the user take action	64	34
23	The material provides simple instructions or examples of how to perform calculations	4	13
24	The material explains how to use the charts, graphs, tables, or diagrams to take actions	8	11
25	The material uses visual aids whenever they could make it easier to act on the instructions	43	23

Each item on the PEMAT was scored on a scale of “agree” = 1, “disagree” = 0 or “Not Applicable (NA)”. Materials marked “Not applicable” are excluded from the denominator

When comparing by topic, the ANOVA revealed the main effect of group assignment [*F* (4, 181) = 12.47, *p* < 0.001, *η*^2^ = 0.21]. Tukey’s multiple comparison test showed that the materials on lifestyle modification scored significantly higher in both understandability and actionability than those on symptoms and treatment options (lifestyle modification vs. signs and symptoms: *M* = 77.6 vs. *M* = 20.4, *p* = 0.03; lifestyle modification vs. treatment options: *M* = 77.6 vs. *M* = 31.2, *p* < 0.001) (Table 3). A comparison of each PEMAT-P item score showed that the material on lifestyle modification scored the highest for almost all items. Multiple comparisons of the PEMAT-P items showed that on most of the actionability items, materials on lifestyle modification had higher mean scores than those on an overview of CKD, signs and symptoms, and treatment options (Supplementary files 3 and 7).

**Table 3:** PEMAT understandability and actionability of web pages by categories

	*n*	%	Understandability			Actionability		
			Mean	SD	*p*-value	mean	SD	*p*-value
Topic								
Overview of CKD	104	55.9	56.8	14.8	< 0.05	31.2	25.8	< 0.05
Signs and symptoms	17	9.1	63.9	14.8		20.4	22.5	
Screening and testing	11	5.9	70.5	13.9		48.5	28.8	
Treatment options	24	12.9	57.5	16.2		31.2	29.7	
Lifestyle modification	30	16.1	76.7	12.3		77.6	17.5	
Source								
Medical institution	64	34.4	57.2	15.4	< 0.05	34	28.4	0.23
Academic institution	17	9.1	53.2	15.2		30.6	29.4	
For-profit company	68	36.6	68.5	15.9		45	34.2	
Governmental organization	19	10.2	61.8	10.1		40	21.4	
Non-profit organization	18	9.7	58.1	18.3		38	31.8	
Target audience								
Patients and their families	135	72.6	61.4	17.2	0.82	62	13.8	0.78
General public	51	27.4	38.3	31.9		39.8	27.3	

One-way ANOVA was performed for comparisons by topic and source. When significant differences were found, Tukey’s multiple comparisons were performed. For comparisons by the audience, an unpaired t-test was used. If there was a significant difference, Kruskal–Wallis multiple comparisons were performed

*SD* standard deviation

The materials created by medical institutions and academic organizations scored lower in understandability than those created by for-profit companies. For actionability, there was no significant difference between any of the sources [*F* (4, 181) = 1.42, *p* < 0.001, *η*^2^ = 0.03] (Table 3). However, multiple comparisons of PEMAT-P items showed that for-profit companies scored higher than governmental agencies on item 25, that is, “The material uses visual aids whenever they could make it easier to act on the instructions” (for-profit companies vs. governmental agencies: *M* = 0.32 vs. *M* = 0, *p* = 0.02) (Supplementary files 4 and 7).

Regarding the audience, there were no significant differences in understandability and actionability (Table 3).

### Quality

The mean GQS was 4.2 (SD 0.9). Regarding topics, the ANOVA revealed the main effect of group assignment [*F* (4, 181) = 4.90, *p* < 0.001, *η*^2^ = 0.098]. Webpages on lifestyle modification were more comprehensive and flowed more naturally than those on CKD overview (lifestyle modification vs. CKD overview: *M* = 4.67 vs. *M* = 3.98, *p* = 0.002). Regarding the source, ANOVA revealed the main effect of group assignment [*F* (4, 181) = 6.69, *p* < 0.001, *η*^2^ = 0.128]. Webpages created by governmental agencies scored lower than those created by for-profit companies or medical institutions (for-profit companies vs. governmental agencies: *M* = 4.47 vs. *M* = 3.63, *p* = 0.002; medical institutions vs. governmental agencies: *M* = 4.39 vs. *M* = 3.63, *p* = 0.004). When compared by audience (patients and their families vs. the general public = 4.28 vs. 4.08, *p* = 0.08), the GQS did not differ between categories (Supplementary files 5 and 7).

### Readability analysis

The mean jReadability score was 2.2 (SD 0.7). This score indicates that the materials can be understood by those who comprehend technical texts and literary works. When compared by topic, the ANOVA revealed a main effect of group assignment [*F* (4, 181) = 3.02, *p* = 0.019, *η*^2^ = 0.062]. The readability scores tended to be higher for webpages focused on lifestyle modification (lifestyle modification vs. overview of CKD: *M* = 2.63 vs. *M* = 2.16, *p* = 0.012; lifestyle modification vs. treatment options: *M* = 2.63 vs. *M* = 2.09, *p* = 0.041). When compared by source, ANOVA revealed the main effect of group assignment [*F* (4, 181) = 2.59, *p* = 0.038, *η*^2^ = 0.054]. Although there were no differences in Tukey’s HSD, the materials produced by for-profit companies tended to score higher than those produced by medical institutions (for-profit companies vs. medical institutions: *M* = 2.38 vs. *M* = 2.08, *p* = 0.11). There was no difference in scores by audience (patients and their families vs. the general public = 2.27 vs. 2.24, *p* = 0.80) (Supplementary file 6).

## Discussion

### Characteristics of overall webpages

One of the key results of our study was the lack of understandable and actionable CKD information online. The webpages had an average understandability and actionability of 61.5% and 38.7%, respectively; approximately 60% and 80% of the materials were not understandable and actionable, respectively. Since inadequate engagement in self-management has been well-documented in CKD [37, 38], the scarcity of materials to support health behaviors makes it challenging for CKD patients.

Similar to previous studies, we found that actionability scores were lower than understandability scores [20, 39–41]. For understandability, the webpages contained several medical terms, and although visual aids were attached, the majority were unclear and simple. In addition, for actionability, a few webpages utilized visual aids to encourage the general public to take action. One study reported that the materials needed to focus on improving the actionability of the advice given and the way the visual aids were presented [20]. Our study reveals that the same issues exist in Japanese webpages.

### Comparison of webpages by categories

The webpages on lifestyle modification were more understandable and actionable than those on signs/symptoms and treatment. Regarding quality and readability, materials on lifestyle modification scored higher than those on the other themes. These materials used fewer medical terms and effectively employed illustrations of desirable lifestyle (e.g., appropriate food intake). A study has reported that patient-centered content is associated with higher-quality materials [17]. However, similar to a previous study [25], these results showed that most websites presented a brief overview of CKD with little information about delaying disease progression. To make more actionable materials for the general public, we recommend webpage authors present not only an overview but also detailed and specific information on CKD. Additionally, webpages must use checklists and flowcharts for topics other than lifestyle modification to assist patients in seeking medical attention and following up at medical facilities.

Regarding understandability, actionability, comprehensiveness, and the natural flow of information, materials created by commercial companies scored higher than those produced by medical institutions and academic organizations. Specifically, commercial companies (mainly pharmaceutical companies) ensured that the materials’ purpose was clearly understood, did not include extra information, and made good use of visual aids. The PEMAT-P showed that medical and academic institutions used medical terminology more frequently and without definitions.

Although previous studies which analyzed English-language materials showed that materials created by public institutions were of higher quality and readability, our study showed contrasting results [41–43]. In Japan, the lack of enforceable guidelines for public and professional institutions, such as the Plain Language Act of 2010 [44], might be a factor affecting the results. Institutional guidelines to improve and standardize the understandability and actionability of health-related materials in Japan must be further developed. The Japanese version of the PEMAT, currently the only reliable and validated indicator for measuring the quality of health information in Japan, has the potential to improve this situation. In addition to developing guidelines, individual healthcare organizations should utilize the expertise of commercial companies to create materials for educating patients about CKD.

### Strengths and limitations

This study has several limitations. First, since this study only focused on Japanese webpages, the generalizability of the results is lacking. Second, the analysis did not include materials that were unavailable on the Internet. Third, the materials were not verified for compliance with evidence-based practice guidelines; however, a board-certified nephrologist checked the contents for any misinformation. Third, for PEMAT-P and GQS, two raters evaluated and calculated IRRs, but we could not completely rule out rating bias. In addition, patient education materials need to be provided by healthcare providers on a regular and ongoing basis, not just at a single point in time. Furthermore, revisions of guidelines and advances in treatment options may change the behavior recommendations of the materials. We, therefore, consider that a longitudinal analysis of the material is critical as part of future studies. Lastly, although prior studies have shown that the PEMAT has been verified for predictive validity through surveys of the general public [19, 28], the items of the PEMAT do not fully reflect the patient’s perspective. Therefore, the opinions of the audience should be evaluated elsewhere. Future research is needed to qualitatively assess how the audience feel about the materials, or how they are likely to take actions recommended in the materials. Despite these limitations, to our knowledge, this study is the first quantitative content analysis of CKD materials widely used in Japan and has important implications. Shared Decision Making (SDM) is not yet well established in clinical practice in Japan. Patients often hesitate to discuss questions or concerns with healthcare professionals during clinical visits. We believe that patient education materials can help bridge the gap between patients and healthcare professionals by providing knowledge and information to patients and facilitating better communication with healthcare professionals. Therefore, understanding the challenges of these materials is essential to improving the quality of care for patients with CKD.

## Conclusion

Although the Internet is a popular source of health information for the general public, we found that CKD information available online was neither understandable nor actionable. They lacked clear, simple visual aids designed to encourage patients to take action. Professional organizations provide poorer quality CKD information with lower actionability than for-profit companies. Furthermore, there is a need to generalize the expertise of commercial companies in preparing information and developing guidelines for communicating health and medical information in an understandable and actionable manner.

### Supplementary Information

Below is the link to the electronic supplementary material.

Supplementary file1 (XLSX 10 KB)

Supplementary file2 (XLSX 13 KB)

Supplementary file3 (XLSX 16 KB)

Supplementary file4 (XLSX 15 KB)

Supplementary file5 (XLSX 12 KB)

Supplementary file6 (XLSX 11 KB)

Supplementary file7 (XLSX 20 KB)
